# Orthotopic Transplantation of Cryopreserved Mouse Ovaries and Gonadotrophin Releasing Hormone Analogues in the Restoration of Function following Chemotherapy-Induced Ovarian Damage

**DOI:** 10.1371/journal.pone.0120736

**Published:** 2015-03-26

**Authors:** Qing Li, Peter Szatmary, Yanyang Liu, Zhenyu Ding, Jin Zhou, Yi Sun, Feng Luo, Yan Wang, Jiang Zhu

**Affiliations:** 1 Department of Oncology, the 452nd hospital of People's Liberation Army, Sichuan, Chengdu, People’s Republic of China; 2 Department of Molecular and Clinical Cancer Medicine, Royal Liverpool University Hospital, University of Liverpool, Liverpool, United Kingdom; 3 West China School of Medince, Sichuan University, Sichuan, Chengdu, People’s Republic of China; 4 Department of Thoracic Oncology, West China Hospital, Sichuan University, Sichuan, Chengdu, People’s Republic of China; 5 Department of Chemotherapy, Sichuan Cancer Hospital, Sichuan, Chengdu, People’s Republic of China; 6 Department of Medical Oncology, Cancer Center and State Key Laboratory of Biotherapy, West China Hospital, Sichuan University, Sichuan, Chengdu, People’s Republic of China; 7 Department of IVF of West China Second Hospital, Sichuan University, Sichuan, Chengdu, People’s Republic of China; Qingdao Agricultural University, CHINA

## Abstract

Therapy advances are constantly improving survival rates of cancer patients, however the toxic effects of chemotherapy drugs can seriously affect patients’ quality of life. In women, fertility and premature ovarian endocrine dysfunction are of particular concern. It is urgently we find methods to preserve or reconstruct ovarian function for these women. This study compares GnRHa treatment with ovarian tissue cryopreservation and orthotopic transplantation in a chemotherapy-induced ovarian damage murine model. 56 inbred Lewis rats were divided into 4 treatment groups: Saline control (group I); cyclophosphamide only (group II); cyclophosphamide plus GnRHa (group III); cyclophosphamide and grafting of thawed cryopreserved ovaries (group IV). Body weight, estrous cycle recovery time, ovarian weight, morphology and follicle count, as well as breeding and fertility were compared among groups. Only group IV was able to restore to normal body weight by the end of the observation period and resumed normal estrous cycles in a shorter time compared to other treatment groups. There was a decrease in primordial follicles in all treatment groups, but group III had the greatest reduction. Although, there was no difference in pregnancy, only one animal littered normal pups in group II, none littered in group III and four littered in group IV. Thus, cryopreservation and orthotopic transplantation of ovarian tissue can restore the fertility of rats subjected to chemotherapy in a manner that is superior to GnRHa treatment. We also observed increased rates of hepatic, splenic and pulmonary haemorrhage in group III, suggesting there may be synergistic toxicity of GnRHa and cyclophosphamide.

## Introduction

Advances in cancer diagnosis and treatment have led to greater numbers of long-term survivors. Use of chemotherapeutic agents, however, can seriously impair endocrine function and fertility due to gonadal toxicity. Chemotherapy induced premature ovarian failure (POF) significantly reduces the quality of life in female survivors and leads to sterility and other health-related problems such as osteoporosis, hot flushes, sleep disturbance, vaginal dryness, and low libido[1–3]. Precocious menopause and irreversible infertility occur in approximately 80%–100% of women subjected to chemotherapy or total body irradiation and bone marrow transplantation [4].

Given the serious consequences young women with cancer face, it is important to aim to improve methods for preservation or restoration of ovarian function.

Different approaches to preserve fertility in women exposed to chemotherapy have been developed and have their particular benefits and drawbacks. Embryo and oocyte preservation technologies, for example, are complex, expensive, and have low success rates [5–7]. Stimulation of ovaries before collection increases estrogen exposure, potentially adding risk in cases of hormone-dependent cancers [8,9]. Minimizing gonadal damage by administering protective drugs during chemotherapy represents an attractive option. Animal experiments have suggested that gonadotropin-releasing hormone analogs (GnRHa) are effective in preventing POF from alkylating agents [10–12]. Although this concept is appealing, there is some debate about the efficacy of GnRHa therapy in humans [13–16]. More importantly, GnRHa treatment alone cannot restore fertility for patients who must accept oophorectomy.

Ovarian tissue cryopreservation and transplantation is a simple, viable, and low-cost treatment option, which can save a large number of follicles, and not only recovers fertility but also restores endocrine function. It can be used for prepubertal girls as well as for women requiring urgent anti-cancer treatment in whom there is insufficient time for ovarian stimulation and oocyte retrieval. Ovarian tissues cryopreservation and autologous transplantation is highly efficient and has been widely studied in recent years and its capability of restoring ovarian function and fertility has been repeatedly documented in animals[17–20]. Donnez et al. reported the world’s first live birth after autologous transplantation of frozen/thawed ovarian tissue, and since then several other similar reports have been published[21–24].

Ovarian tissue cryopreservation methods mainly consist of slow-freezing and vitrification. Slow-freezing can result in intracellular ice crystal damage and is complicated and costly. Vitrification uses faster freezing rates and avoids formation of intracellular ice crystal in addition to being more efficient and convenient[25–28]. A novel method termed needle immersed vitrification (NIV) has been described in previous studies conducted by our unit[29]. NIV proved to be advantageous in cryopreserving both normal mouse and human ovarian tissue [29,30]. However, studies on ovarian cryopreservation and transplantation only rarely use the chemotherapy-induced ovarian damage model. The efficiency and safety of ovary cryopreservation and transplantation would make it an attractive option to restore ovarian function following chemotherapy. In this study, we aim to evaluate the ability of ovarian cryopreservation by NIV and orthotopic transplantation to restore ovarian function following chemotherapy-induced ovarian damage and compare this to GnRHa treatment.

## Materials and Methods

### Animals

All animals (inbred Lewis rats) were purchased from Beijing HFK Bioscience CO.,LTD. A total of 72 mature virginal females rats (8–9 weeks old) weighing 200–220g were examined. Vaginal smears were obtained daily and 70 rats demonstrating at least 2 normal consecutive estrous cycles were included in the experiments. 10 males (8–10 weeks old) were used for mating studies. All rats were housed in specific pathogen free facilities, were fed rat chow and water *ad libitum* and kept in transparent cages with 2–3 rats/cage at controlled temperature (22–24°C) and light (12 h light, 12 h dark). Approval by the Institutional Animal Care and Use Committee, Sichuan University has been granted.

### Chemicals

Cyclophosphamide for injection (cyclophosphamide, Jiang Su Heng Rui Medicine CO.,LTD, Jiangsu, China) was prepared daily in sterile 0.9% NaCI solution at a concentration of 2 mg/ml[31]. Luprorelin Acetate Microspheres for injection (Luprorelin, Shanghai Livzon Medicine CO.,LTD, Shanghai, China) were prepared in suspension at a final concentration of 1.875 mg/ml according to the instructions.

### Experimental design

Donor ovaries were collected from 14 rats selected at random. The remaining 56 rats were divided into 4 treatment groups of 14 animals each. Group I (control) received daily i.p. injections of 2 ml 0.9% NaCl for 21 days. Group II (cyclophosphamide only) received a loading dose of cyclophosphamide (50 mg/kg) followed by daily i.p. injections of 10 mg/kg[31]. Group III (GnRHa) received the same cyclophosphamide treatment as Group II as well as a single s.c. injection of 140 μg Leuprorelin [11, 31] (calculated to release 5 μg/day) 1 week prior to commencing cyclophosphamide therapy. Group IV (cryopreservation and orthotopic transplantation) received the same cyclophosphamide treatment as groups II and III, followed by grafting of thawed cryopreserved ovarian tissues into the ovarian bursae.

Vaginal smears were examined daily in all groups from 1 week prior to commencing drug treatment to four weeks following treatment cessation. Body weight was also assessed daily as a marker of general animal health. 2 animals from each group were sacrificed and had their ovaries removed for histological examination. Liver, lungs and spleen were also examined histologically at the 2 and 4 week timepoints. The remaining rats were then paired with sexually mature, fertile males.

### Freezing and thawing

Ovaries from 9–10 weeks old rats killed by cervical dislocation were aseptically removed and collected in Leibovitz medium (L-15, Gibco, Grand Island, NY, USA) supplemented with 10% fetal bovine serum (FBS, Gibco), 50 mg/ml of streptomycin and 50 IU/ml of Penicillin (Gibco). The ovaries were cleaned of surrounding tissues and sectioned into 3x3x3mm cubes. This dissection was performed in Leibovitz (L15) medium on ice in less than one hour. The vitrification protocol (NIV) was as described by Wang *et al*.[29] Briefly, a special carrier or ‘acupuncture needle’ (Cloud & Dragon Medical Device Co. Ltd, China) was used; the dimensions of this needle (0.18x13 mm), allowed several tissue samples to be held simultaneously. The ovarian tissue samples were dehydrated using a two-step regimen: (i) immersed in an equilibration solution consisting of 7.5% (v/v) ethylene glycol (EG, Sigma-Aldrich) and 7.5% (v/v) dimethyl sulfoxide (DMSO, Sigma-Aldrich) in Leibovitz medium supplemented with 20% FBS for 10 min at room temperature followed by (ii) transfered to a vitrification solution consisting of 15% EG, 15% DMSO and 0.5 M sucrose (Sigma-Aldrich, St Louis, MO, USA) and immersed for 2 min. The dehydrated ovarian tissues were then placed on an aseptic absorbent gauze to remove the remaining vitrification solution then immersed into liquid nitrogen directly. By using the acupuncture needle all the samples can be exposed to cryoprotectants and liquid nitrogen simultaneously and under the same conditions.

For thawing, the needles holding ovarian tissue were taken out of the vial using forceps and quickly immersed into 1 M sucrose solution, which had been pre-warmed at 37°C for 5 min. They were serially transferred into 0.5 M and 0.25 M sucrose solution for 5 min each and were incubated in Leibovitz medium supplemented with 20% FBS for 20 min at 37°C with 5% CO2 before re-implantation.

### Transfer to the ovarian bursa

Freeze–thawed ovaries from donors were transplanted into the bursae of group IV rats as per Liu *et al*.[32]. Briefly, the recipients were anesthetized by i.p. injection of 10% chloral hydrate (0.004 ml/g) and the laparotomies performed under strictly aseptic conditions. The ovarian bursae were accessed through a small dorsally horizontal incision, which was extended just below the fat pad, and the bursal membrane was reflected over the ovary. A pair of fine watchmaker’s forceps was used to grip the hilum, and a second pair was used to sever the native ovary from the hilum and thoroughly remove residual ovarian tissues. Pressure was applied to control bleeding. The donor ovary was placed within the bursal cavity, with the bursal membrane replaced and closed with 9–0 nylon sutures before returning it to the peritoneal cavity. Both ovaries of the recipient animal were removed and replaced with donor ovaries.

### Vaginal smears

Vaginal smears were taken daily from all female rats. The vaginal wall of each rat was scraped gently using a sterile swab soaked with saline and the cells were smeared onto a clean glass slide. The air-dried smears were stained with an aqueous solution of 0.1% methylene blue. The stage of the estrous cycle was determined from the cell types observed on the smears[33].

### Mating and pregnancy evaluation

Following pairing of animals, successful mating was defined by the presence of spermatozoa on microscopic analysis of the smear, or that of a vaginal plug. Following successful mating, females were separated from males and weighed daily for 15 days to monitor weight gains indicating pregnancy. If no vaginal plug was seen, females were left in continuous pairings with males for 7 days and then weighed on days 15–21. The date of birth and number of pups in each litter was recorded. The pups were removed from their parents 3–4 days after birth and euthanized.

### Histology

Mouse ovaries and vital organs from different groups were fixed in 10% neutral-buffered formalin solution for 24 h at 4°C, dehydrated, paraffin-embedded, and serially sectioned at every 5 μm. The sections were mounted on glass slides and stained with hematoxylin and eosin (H&E) prior to morphological examination. Follicular counts were taken from every fifth section of each ovary. To avoid counting follicles more than once, only follicles with a visible nucleus were counted. Follicles were classified according to the criteria suggested by Myers *et al*.[34] Primordial follicles were defined as an oocyte surrounded by a single layer of flattened granulose cells; primary follicles with one layer of cuboidal granulose cells surrounding the oocyte; secondary follicles as those the oocytes surrounded by two or more layers of cuboidal granulosa cells without an antrum and antral follicles as those with an antral cavity. Only normal follicles were counted and had to contain intact granulosa cell layers, occasional isolated granulosa cells with pyknotic nuclei and unfragmented oocytes with a germinal vesicle [32].

### Statistical analysis

Parametric data are expressed as mean±SD. Analysis of variances (ANOVA) was used to assess differences between the groups, a Kaplan-Meier event analysis and log-rank test was used to compare estrous cycle recovery time and a Chi-squared test was used to compare the proportion of fertile females. Differences were considered significant when *P* <0.05.

## Results

### Body weight

**Fig 1:**
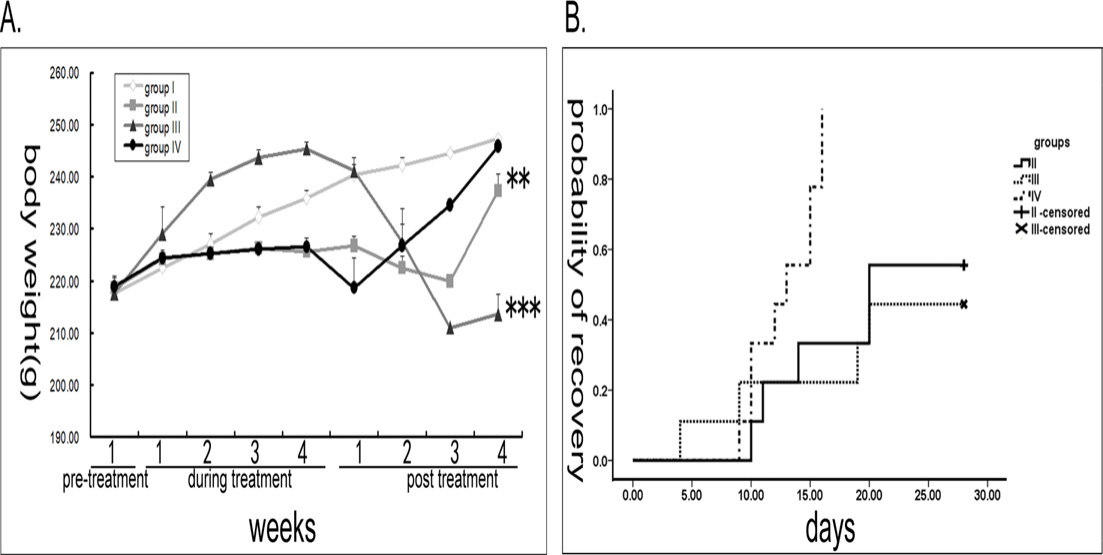
Body weight changes and estrous cycle recovery. (A) Changes in mean body weight in experimental groups. ** p<0.01, *** p<0.001. (B) Recovery of estrous cycle in different experimental groups following cyclophosphamide treatment cessation.

There was no significant difference in body weight between the different groups before treatment (*P*>0.05). The mean body weight of group I gradually increased throughout the experiment. Animals receiving cyclophosphamide, but no GnRHa (II and IV) maintained a steady weight which was significantly lower compared to control animals at the end of the cyclophosphamide period (I) (*P* <0.05), due to the weight gain of the control group. Group IV animals, however, lost weight immediately following surgery, but then rapidly gained weight until their final weights were indistinguishable from control by the end of the four week observation period (*P* = 0.580). The GnRHa group (III), showed very significant (*P* <0.001) weight gain compared to control (I), however, also rapidly lost immediately following treatment cessation. The body weight of groups II and III was significantly lower than control by the end of the observation period (*P* <0.005) (Fig. 1A).

### Estrous cycle

Cyclic changes in vaginal cytology were observed in all of the treated groups. Animals in group I had regular estrous cycles throughout the duration of the observation period. Following chemotherapy (cyclophosphamide), rats in group II developed irregular cycles with prolonged and more frequent estrus periods. All rats were in persistent estrus by the end of the treatment period. Following treatment cessation, 5 out of the 9 cyclophosphamide-only rats gradually recovered normal estrous cycles, the other four remained in persistent estrus until the end of the observation period. The median estrous cycle recovery time was 20 d (95%CI:11.2–28.8). Group III was in persistent diestrus after start of Luprorelin even during the time of cyclophosphamide. Following treatment cessation, the rats began to recover normal cycles and after four weeks, four were cycling regularly with the other five still in persistent diestrus. Median estrous cycle recovery time was 28 d. In group IV, the estrous cycles changed similarly to group II during the period of cyclophosphamide. However, after surgery the rats gradually went into sustained diestrus and with growth of the grafts the rats gradually resumed normal estrous cycles. By the end of the observation period, all rats in group IV appeared to have normal estrous cycles. Median estrous cycle recovery time was 13d (95%CI:10.1–15.9). Recovery to normal estrous cycles was significantly quicker in group IV compared to group II and group III (*P*<0.05, there was no significant difference between group II and group III (*P* = 0.776) (Fig. 1B).

### Ovarian morphology and weight

**Fig 2:**
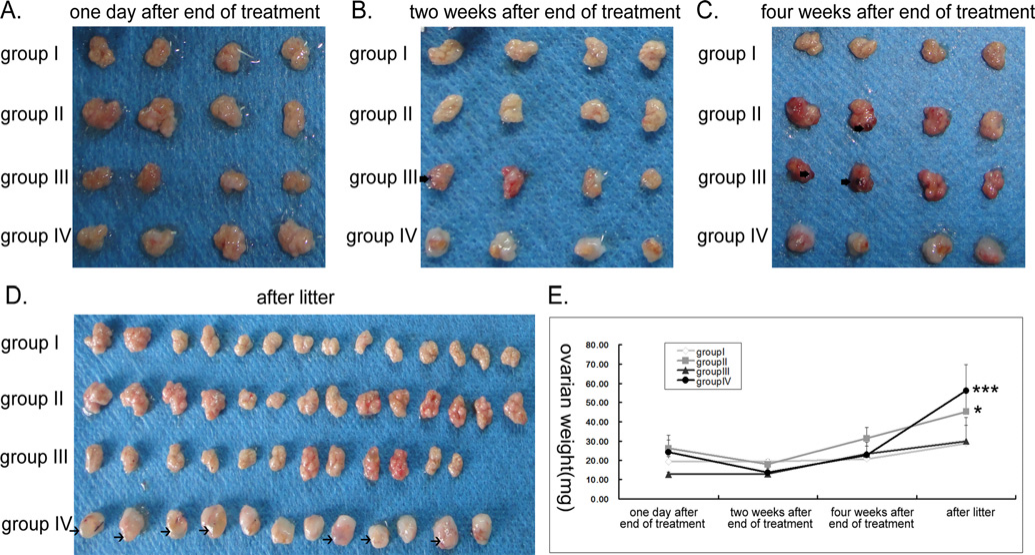
Ovarian morphology and weight at different time points following treatment cessation. Ovarian morphology were compared between different groups at: (A) one day after end of treatment (2 rats), (B) two weeks after end of treatment (2 rats), the black thick arrow points to the black blot on the ovarian surface (C) four weeks after end of treatment (2 rats), the black thick arrow points to the black blot on the ovarian surface (D) after litter, the thin arrow points to the transparent follicles on the ovarian surface (7 rats in group I and group II, 6 rats in group III and group IV). (E) Ovarian weight at different time points. * p<0.05, *** p<0.001.

Ovaries were markedly darker and demonstrated black dots on their surface in weeks 2 and 4 in group III and week 4 in group II (Fig. 2BC). This was not seen at all in group IV where the appearance of transparent follicles on the surface of ovaries could clearly be observed after litter (Fig. 2D). Following treatment cessation, the ovaries of cyclophosphamide treated but no GnRHa animals (II and IV) were slightly heavier and larger, although this was not significant. On the other hand, weight of ovaries in group III was lower than all other groups. At 2 and 4 weeks following treatment cessation, there was no significant difference in ovarian weight between the groups. However after litter, the weights of ovaries in groups II (*P* = 0.018) and IV (*P*<0.001) were significantly greater than control (Fig. 2E).

### Histological examination of the ovaries

**Fig 3:**
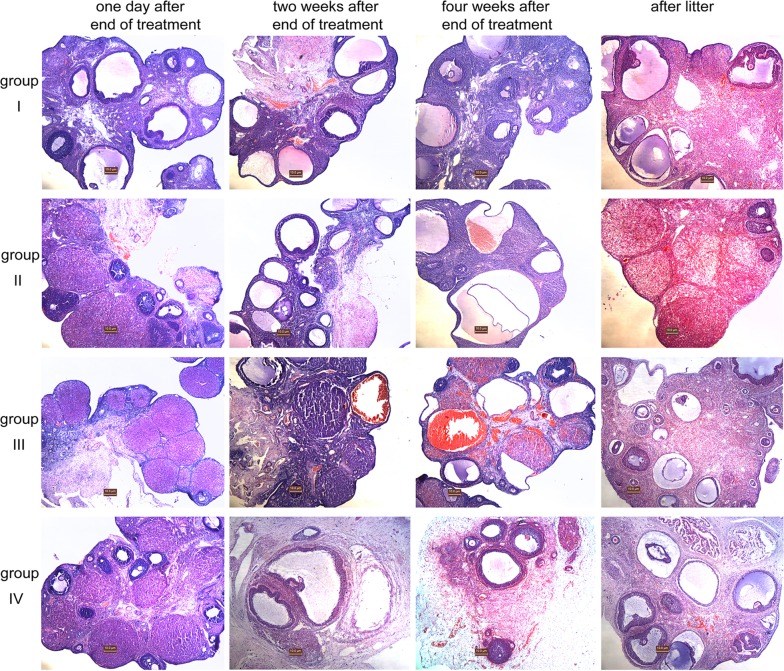
Histological examination of the ovaries at different time points. Morphological representation of the ovaries of different experimental groups at different time points stained by H&E. (original magnification ×50, Bar = 10 um).

Histological examination of the ovaries at low magnification showed that one day after the end of cyclophosphamide, there was a higher proportion of corpora lutea in ovaries of animals in the treated groups compared to control group, this phenomenon was more obviously in group III. Throughout the four-week post cyclophosphamide observation period, follicular cavity hemorrhage could be seen in weeks 2 and 4 in group III and week 4 in group II, which was more obviously in group III by the end of the fourth week. But this was not seen at all in group IV. After litter, the morphological presentation of the ovarian tissue in group IV was similar to the control group. However, fibers surrounding the aging ovarian tissues could be observed in group II, and in group III, there was a higher proportion of secondary follicles (Fig. 3).

### Follicle count

**Fig 4:**
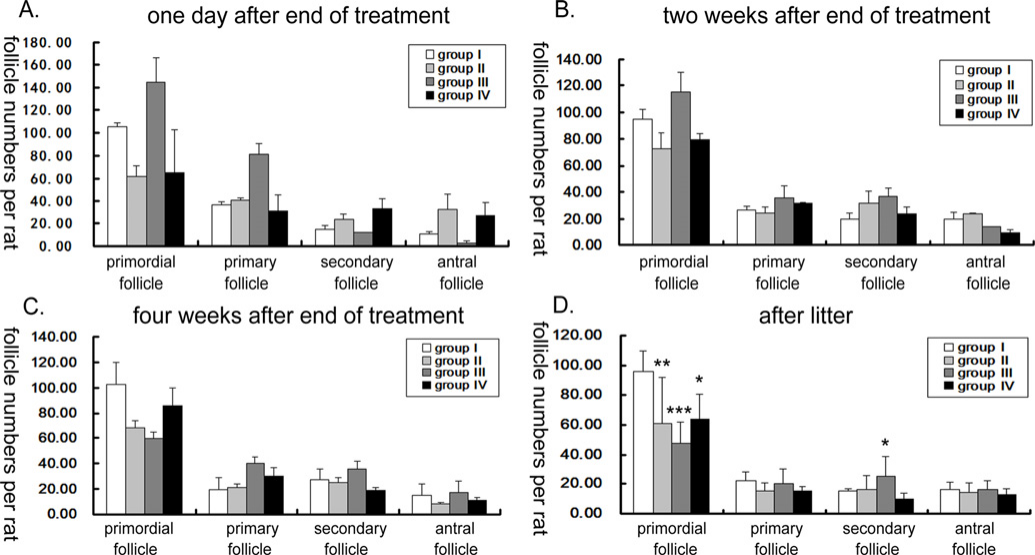
Follicle count at different time points. Morphologically normal follicles in the different stages of development were compared between different groups at: (A) one day after end of treatment, (B) two weeks after end of treatment, (C) four weeks after end of treatment, (D) after litter. * p<0.05, ** p<0.01, *** p<0.001.

One day after the end of treatment, there were reduced numbers of primordial and primary follicles in group II and IV, but greater numbers of secondary and antral follicles. However, In the GnRHa group (III), there were greater numbers of primordial and primary follicles but a marked reduction in secondary and antral follicles (Fig. 4A). Over the next four weeks, the number of primordial follicles remained significantly lower in group II; the number of antral follicles increased in group III with a reduction of primordial follicles; group IV demonstrated a gradually increased number of antral follicles in grafted tissues throughout the observation period after cyclophosphamide treatment (Fig. 4BC). After litter, the number of the primordial follicles showed significantly lower in all the treated groups, however, group III decreased more obviously. On the other hand, in group III, there was a significant increase in the secondary follicles (*P*<0.05) (Fig. 4D).

### Fecundity

Mating began 4 weeks after grafting. Twenty-one days after mating, the offspring from each animal were counted. Table 1 is a summary of observed fertility and fecundity. Six out of seven rats became pregnant in group I with all pups born live and apparently normal. The mean litter size was 5.43±3.74 pups. Not all females that mated were fertile in treated groups. In group II, five out of eight rats became pregnant but only one littered eight normal pups. One rat died after suffering a miscarriage on day thirteen after mating and another ate all pups in the litter. The remaining two were seen to gain weight after mating, but the weight did not continue to increase after a few days, suggesting pseudopregnancy. This phenomenon was also observed in group III with five out of six rats becoming pregnant after mating, but weight gain only occurring in the early stage of pregnancy; none littered. Finally, in group IV four out of six rats became pregnant after mating with a mean litter size of 2.33±2.58 pups.

**Table 1:** Fecundity of the rats.

Group	I	II	III	IV
No. of rats	7	8	6	6
No. of pregnancy(%)	6(85.71)	5(62.50)	5(83.3)	4(66.67)
No. of litter(%)	6(100.00)	1(20.00)	0(0.00)	4(100.00)
No. of pups	38	8	0	14
Pups/group(range)	5.43±3.74(0–11)	1.00±2.83**(0–8)	0.00±0.00** (/)	2.33±2.58(0–6)

** p<0.01, compared with group I.

## Discussion

We have demonstrated for the first time that cryopreservation and orthotopic transplantation of ovarian tissue can restore the fertility of rats subjected to chemotherapy. We have also failed to demonstrate any protective effect of GnRHa on fertility loss in this model; indeed we observed a possible synergistic toxic effect of GnRHa with cyclophosphamide. It is important to note that we observed macroscopic and microscopic haemorrhage in lung, liver and spleen of animals in the GnRHa group and that this was more pronounced than any of the other groups (see S1 Fig.). This may relate to the relatively high doses of cyclophosphamide used in these experiments, however these are in line with other experiments we have conducted. Moreover, we wanted to use a slightly higher dose to ascertain the power of this technique in restoring fertility, even for harsher chemotherapy regimens.

Bokser *et al*. [10] reported the rats treated with the cyclophosphamide and GnRHa showed an augmentation in body weight. We have also demonstrated that while the GnRHa Leuprorelin protects from weight loss during cyclophosphamide and indeed led to weight gain in our experiments. However, the group treated with the cyclophosphamide only or cyclophosphamide and GnRHa showed rapidly weight loss after therapy cessation, and the weight loss in the combination treatment group was more significant, which was similar to Letterie *et al*.’s results [35]. Only orthotopic transplantation of cryopreserved ovarian tissue was able to restore normal weight by the end of our observation period (4 weeks following cessation of cyclophosphamide).

It is well known that the estrous cycle can reflect ovarian function. It has been reported rats receiving cyclophosphamide only or in combination with a GnRHa would stop cycling and go into persistent diestrus [36]. In this study, we indeed observed changes in the cycles of all of the treated groups, and rats treated with the cyclophosphamide/GnRHa combination went into persistent diestrus. Rats in group II and group IV, however, went into persistent estrus during the treatment.

Prior studies reported that rats could restore regular estrus cycles after the end of cyclophosphamide treatment [31, 36]. In our study, group IV resumed a normal estrous cycle in a shorter time compared to all other treatment groups. Notably, all animals in this group returned to normal cycles during the observation period. 4/9 rats in the cyclophosphamide only group (II) and 5/9 rats in the combination therapy group (III) did not resume normal estrous cycles in the observed period of time. This may relate to a slightly larger dose of cyclophosphamide used in our study, but may also reflect on the length of the observation period. Perhaps all animals would have resumed normal cycles given a prolonged observation period. In any case, it is notable that ovarian tissue preservation and transplantation is superior to GnRHa combination therapy in this setup.

In our study, in the early end of treatment, the rats treated with cyclophosphamide only went into persistent estrus, showed a trend for increase in ovarian weight and large follicles but a reduced number of primordial and primary follicles. After litter, the number of primordial follicles remained significantly lower and the weights of ovaries remained significantly greater in this group, however, fibers surrounding the aging ovarian tissues were observed. Generally, the most mitotically active cells are the most sensitive to cyclophosphamide toxicity. One can, therefore, visualize cyclophosphamide-related ovarian toxicity as a destruction of medium to larger follicles, which then induces maturation of smaller follicles thereby again leading to their destruction. Given the fixed, limited store of follicles in an ovary thus, it seems likely that a longer duration of therapy with cyclophosphamide might reduce the number of small follicles and eventually produce ovarian failure. In current study, the duration of cyclophosphamide was limited, cyclophosphamide’s damaging effects on the growing follicles attenuated from the end of treatment, secondary follicles and antral follicles gradually recover to normal, but the consumption of primordial follicles could not be replenished. Finally, the aging ovaries were of greater weight but showed a significant reduction in primordial follicles.

In this model, use of a GnRHa returns the ovaries to a quiescent state, limiting the maturation of follicles and thereby limiting cyclophosphamide toxicity. This was indeed what we observed in the early end of treatment: the rats treated with cyclophosphamide and GnRHa went into persistent diestrus, showed a trend for smaller ovary and a larger number of immature follicles. Then, effect of GnRHa ceased, along with the ovarian weight and larger follicles increased, a simultaneous decrease in number of primordial follicles was observed. After litter, the number of primordial follicles in this group even lower than that in the cyclophosphamide alone group, while the secondary follicles increased, ovarian biopsy also seen more secondary follicles. It was as if there being a ‘brake’ on follicular maturation, on removal of this ‘brake’, the larger pool of smaller follicles all appeared to mature simultaneously, however, leading to an increased number of secondary follicles observed four weeks after cyclophosphamide cessation. The fact that this increased number of follicles resulted in a similar number of pregnancies to both the control and ovarian transplant group, but did not translate to a similar number of live births suggests that these follicles may still have suffered genetic damage; not enough to undergo cell death, but enough to prevent viable pregnancies. Alternatively cessation of GnRHa therapy at the end of cyclophosphamide may have been too early and future studies should consider a ‘wash out period’ where GnRHa therapy is continued for a period of time after cyclophosphamide cessation, to hold back follicles until the last traces of cyclophosphamide agent have been metabolized and cleared.

There are a number of safety concerns arising from our experiments. Notably, mortality was similar in all groups. During the experiment, one of the control animals died on day 30 after termination of treatment, the cause of death was not clear. One rat in group II died after delivering a stillbirth on the thirtieth day after mating. Two animals in group III died on days 18 and 39 after completion of therapy with very low body weights of 197g and 189g. Two rats died in group IV: one on day 8 after surgery due to intestinal obstruction, another on day 14 due to diethyl ether overdose during the second blood collection process. Examination of ovarian tissues and vital organs revealed that two weeks following cyclophosphamide, the liver edge of rats in group II was black; histologically this correlated with haemorrhage at the liver edge. This effect was more prominent in group III, where ovaries demonstrated black dots on their surface and the whole liver was black and histology confirmed diffuse hemorrhage (Fig. 2B, Fig. 3 and S1A Fig.). Four weeks following treatment, haemorrhage was no longer restricted to the ovaries and liver, but could also be observed in the spleen (groups III > II, Fig. 2C, Fig. 3 and S1A Fig.). After litter, no hepatic haemorrhage was seen, but splenic haemorrhage persisted in group III. Necropsy of animals that died in group III with low body weights revealed macroscopic and microscopic haemorrhage of liver, spleen and lungs (S1B Fig.) as well as vacuolar degeneration at the liver edges (S1C Fig.). While it is to be expected that any interventional procedure carries associated risk, even a mortality, it is not clear why the degree of ovaries, hepatic, splenic and even pulmonary haemorrhage should be so much more marked in the GnRHa group. This finding raises a particular concern that there may be synergistic toxicity of cyclophosphamide and luprorelin and this certainly warrants further in depth investigation.

There are a number of limitations to our study. Firstly, we decided not to measure hormone levels at different time points as we were primarily interested in the ability of our technique to restore fertility. Nevertheless, future studies to investigate whether and in how far ovarian endocrine function can be restored with this technique would be of interest. Secondly, we chose to transplant the grafts immediately after the end of chemotherapy, which is not consistent with normal clinical practice where you would expect a period of recovery prior to any further surgery. However, this meant the rats would have been in a worse state than if they had been allowed to recover first and indeed there may still have been some drug toxicity effect on the newly transplanted grafts. This makes the ability of the grafts to restore fertility and weight even more impressive.

## Conclusions

Fertility preservation is an increasingly recognised issue in cancer patients and of growing importance. The role of GnRHa as ovarian protection agents remains an unproven entity, as there is still significant uncertainty over its mechanism, effectiveness and safety. Ovarian tissue cryopreservation and orthotopic transplantation, although still experimental, has been shown to restore fertility and may even protect ovarian endocrine function. As such, oncologists and reproductive medicine specialists should work together to make it a safe and effective option offered to all young women diagnosed with cancer.

## Supporting Information

S1 Fig Morphological presentation of the organs at different time points. (A) Morphological presentation of the liver and spleen stained by H&E. (original magnification ×200, Bar = 10 um). (B) The general morphology and the histological examination of lung of the dead rat in group III. The black thick arrow presented the black blot on the lung. (original magnification ×200, Bar = 10 um). (C) The histological examination of the liver of the dead rat in group III. The black thin arrow presented the vacuolar degeneration (original magnification ×200, ×400, Bar = 10 um).
(TIF)

## References

[1] Walshe JM, Denduluri N, Swain SM. Amenorrhea in premenopausal women after adjuvant chemotherapy for breast cancer. Journal of clinical oncology: official journal of the American Society of Clinical Oncology. 2006; 24: 5769–5779. 17130515 10.1200/JCO.2006.07.2793

[2] Schover LR. Premature ovarian failure and its consequences: vasomotor symptoms, sexuality, and fertility. Journal of clinical oncology: official journal of the American Society of Clinical Oncology. 2008; 26: 753–758. 18258983 10.1200/JCO.2007.14.1655

[3] Ruddy KJ, Partridge AH. Fertility (male and female) and menopause. Journal of clinical oncology: official journal of the American Society of Clinical Oncology. 2012; 30: 3705–3711. 23008319 10.1200/JCO.2012.42.1966

[4] Wallace WH, Thomson AB, Saran F, Kelsey TW. Predicting age of ovarian failure after radiation to a field that includes the ovaries. International journal of radiation oncology, biology, physics. 2005; 62: 738–744. 15936554 10.1016/j.ijrobp.2004.11.038

[5] Dolmans MM, Demylle D, Martinez-Madrid B, Donnez J. Efficacy of in vitro fertilization after chemotherapy. Fertility and sterility. 2005; 83: 897–901. 15820797 10.1016/j.fertnstert.2004.08.035

[6] Oktay K, Cil AP, Bang H. Efficiency of oocyte cryopreservation: a meta-analysis. Fertility and sterility. 2006; 86: 70–80. 16818031 10.1016/j.fertnstert.2006.03.017

[7] Imai A, Sugiyama M, Furui T, Tamaya T, Ohno T. Direct protection by a gonadotropin-releasing hormone analog from doxorubicin-induced granulosa cell damage. Gynecologic and obstetric investigation. 2007; 63: 102–106. 17028438 10.1159/000096062

[8] Pike MC, Spicer DV, Dahmoush L, Press MF. Estrogens, progestogens, normal breast cell proliferation, and breast cancer risk. Epidemiologic reviews. 1993; 15: 17–35. 8405201 10.1093/oxfordjournals.epirev.a036102

[9] Key T, Appleby P, Barnes I, Reeves G. Endogenous Hormones and Breast Cancer Collaborative Group. Endogenous sex hormones and breast cancer in postmenopausal women: reanalysis of nine prospective studies. Journal of the National Cancer Institute. 2002; 94: 606–616. 11959894 10.1093/jnci/94.8.606

[10] Bokser L, Szende B, Schally AV. Protective effects of D-Trp6-luteinising hormone-releasing hormone microcapsules against cyclophosphamide-induced gonadotoxicity in female rats. British journal of cancer. 1990; 61: 861–865. 2142603 10.1038/bjc.1990.192 PMC1971695

[11] Ataya K, Ramahi-Ataya A. Reproductive performance of female rats treated with cyclophosphamide and/or LHRH agonist. Reproductive toxicology. 1993; 7: 229–235. 8318754 10.1016/0890-6238(93)90229-z

[12] Ataya K, Rao LV, Lawrence E, Kimmel R. Luteinizing hormone-releasing hormone agonist inhibits cyclophosphamide-induced ovarian follicular depletion in rhesus monkeys. Biology of reproduction. 1995; 52: 365–372. 7711205 10.1095/biolreprod52.2.365

[13] Beck-Fruchter R, Weiss A, Shalev E. GnRH agonist therapy as ovarian protectants in female patients undergoing chemotherapy: a review of the clinical data. Human reproduction update. 2008; 14: 553–561. 10.1093/humupd/dmn041 18820006

[14] Peccatori F, Demeestere I. GnRH analogue for chemotherapy-induced ovarian damage: too early to say? Fertility and sterility. 2009; 92: e33; author reply e4. 10.1016/j.fertnstert.2009.06.002 19646600

[15] Levine J, Canada A, Stern CJ. Fertility preservation in adolescents and young adults with cancer. Journal of clinical oncology: official journal of the American Society of Clinical Oncology. 2010; 28: 4831–4841. 20458029 10.1200/JCO.2009.22.8312

[16] Rugo HS, Rosen MP. Reducing the long-term effects of chemotherapy in young women with early-stage breast cancer. JAMA: the journal of the American Medical Association. 2011; 306: 312–314. 21771995 10.1001/jama.2011.1019

[17] Candy CJ, Wood MJ, Whittingham DG. Restoration of a normal reproductive lifespan after grafting of cryopreserved mouse ovaries. Human reproduction. 2000; 15: 1300–1304. 10831559 10.1093/humrep/15.6.1300

[18] Salle B, Demirci B, Franck M, Berthollet C, Lornage J. Long-term follow-up of cryopreserved hemi-ovary autografts in ewes: pregnancies, births, and histologic assessment. Fertility and sterility. 2003; 80: 172–177. 12849820 10.1016/s0015-0282(03)00554-5

[19] Almodin CG, Minguetti-Camara VC, Meister H, Ferreira JO, Franco RL, Cavalcante AA, et al Recovery of fertility after grafting of cryopreserved germinative tissue in female rabbits following radiotherapy. Human reproduction. 2004; 19: 1287–1293. 15117903 10.1093/humrep/deh246

[20] Bosch P, Hernandez-Fonseca HJ, Miller DM, Wininger JD, Massey JB, Lamb SV, et al Development of antral follicles in cryopreserved cat ovarian tissue transplanted to immunodeficient mice. Theriogenology. 2004; 61: 581–594. 14662154 10.1016/s0093-691x(03)00244-9

[21] Donnez J, Dolmans MM, Demylle D, Jadoul P, Pirard C, Squifflet J, et al Livebirth after orthotopic transplantation of cryopreserved ovarian tissue. Lancet. 2004; 364: 1405–1410. 15488215 10.1016/S0140-6736(04)17222-X

[22] Andersen CY, Rosendahl M, Byskov AG, Loft A, Ottosen C, Dueholm M, et al Two successful pregnancies following autotransplantation of frozen/thawed ovarian tissue. Human reproduction. 2008; 23: 2266–2272. 10.1093/humrep/den244 18603535

[23] Donnez J, Squifflet J, Jadoul P, Demylle D, Cheron AC, Van Langendonckt A, et al Pregnancy and live birth after autotransplantation of frozen-thawed ovarian tissue in a patient with metastatic disease undergoing chemotherapy and hematopoietic stem cell transplantation. Fertility and sterility. 2011; 95: 1787 e1–4. 10.1016/j.fertnstert.2010.11.041 21145049

[24] Revelli A, Marchino G, Dolfin E, Molinari E, Delle Piane L, Salvagno F, et al Live birth after orthotopic grafting of autologous cryopreserved ovarian tissue and spontaneous conception in Italy. Fertility and sterility. 2013; 99: 227–230. 10.1016/j.fertnstert.2012.09.029 23102860

[25] Chen SU, Lien YR, Chao K, Lu HF, Ho HN, Yang YS. Cryopreservation of mature human oocytes by vitrification with ethylene glycol in straws. Fertility and sterility. 2000; 74: 804–808. 11020527 10.1016/s0015-0282(00)01516-8

[26] Chen SU, Lien YR, Chen HF, Chao KH, Ho HN, Yang YS. Open pulled straws for vitrification of mature mouse oocytes preserve patterns of meiotic spindles and chromosomes better than conventional straws. Human reproduction. 2000; 15: 2598–2603. 11098033 10.1093/humrep/15.12.2598

[27] Migishima F, Suzuki-Migishima R, Song SY, Kuramochi T, Azuma S, Nishijima M, et al Successful cryopreservation of mouse ovaries by vitrification. Biology of reproduction. 2003; 68: 881–887. 12604638 10.1095/biolreprod.102.007948

[28] Yeoman RR, Wolf DP, Lee DM. Coculture of monkey ovarian tissue increases survival after vitrification and slow-rate freezing. Fertility and sterility. 2005; 83 Suppl 1: 1248–1254. 15831299 10.1016/j.fertnstert.2004.11.036

[29] Wang Y, Xiao Z, Li L, Fan W, Li SW. Novel needle immersed vitrification: a practical and convenient method with potential advantages in mouse and human ovarian tissue cryopreservation. Human reproduction. 2008; 23: 2256–2265. 10.1093/humrep/den255 18614614

[30] Xiao Z, Wang Y, Li L, Luo S, Li SW. Needle immersed vitrification can lower the concentration of cryoprotectant in human ovarian tissue cryopreservation. Fertility and sterility. 2010; 94: 2323–2328. 10.1016/j.fertnstert.2010.01.011 20189561

[31] Ataya KM, McKanna JA, Weintraub AM, Clark MR, LeMaire WJ. A luteinizing hormone-releasing hormone agonist for the prevention of chemotherapy-induced ovarian follicular loss in rats. Cancer research. 1985; 45: 3651–3656. 3926307

[32] Liu L, Wood GA, Morikawa L, Ayearst R, Fleming C, McKerlie C. Restoration of fertility by orthotopic transplantation of frozen adult mouse ovaries. Human reproduction. 2008; 23: 122–128. 17993475 10.1093/humrep/dem348

[33] Rugh R. The mouse: its reproduction and developmented. Oxford England; New York: Oxford University Press; 1990.

[34] Myers M, Britt KL, Wreford NG, Ebling FJ, Kerr JB. Methods for quantifying follicular numbers within the mouse ovary. Reproduction. 2004; 127: 569–580. 15129012 10.1530/rep.1.00095

[35] Letterie GS. Anovulation in the prevention of cytotoxic-induced follicular attrition and ovarian failure. Human Reproduction. 2004; 19:831–837. 15033951 10.1093/humrep/deh120

[36] Ghosha S, Misrob M, Dasa UB, Maiti R, Debnath JM, Ghosh D. Effect of human chorionic gonadotrophin coadministration on ovarian steroidogenic and folliculogenic activities in cyclophosphamide treated albino rats. Reprod Toxicol. 2001; 15:221–225. 11297880 10.1016/s0890-6238(01)00114-9

