# Supplementary figures and images for: Orthotopic Transplantation of Cryopreserved Mouse Ovaries and Gonadotrophin Releasing Hormone Analogues in the Restoration of Function following Chemotherapy-Induced Ovarian Damage
Source: PLoS One. 2015 Mar 26;10(3):e0120736. doi: 10.1371/journal.pone.0120736 (PMC4374936; doi:10.1371/journal.pone.0120736)

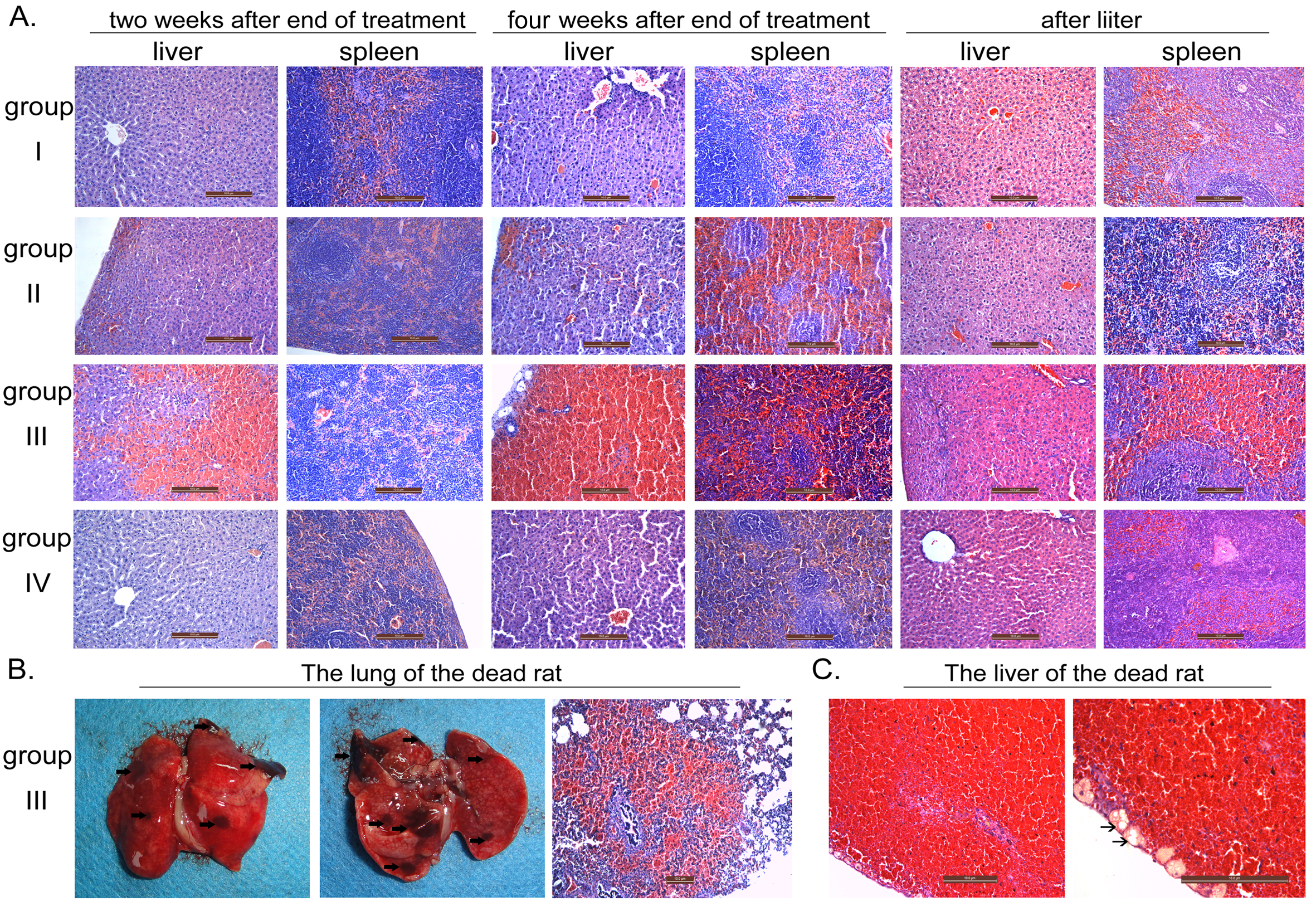

Supplement: S1 Fig — (A) Morphological presentation of the liver and spleen stained by H&E. (original magnification ×200, Bar = 10 um). (B) The general morphology and the histological examination of lung of the dead rat in group III. The black thick arrow presented the black blot on the lung. (original magnification ×200, Bar = 10 um). (C) The histological examination of the liver of the dead rat in group III. The black thin arrow presented the vacuolar degeneration (original magnification ×200, ×400, Bar = 10 um). (TIF) [file pone.0120736.s001.tif]
